# Type II diabetes mellitus and hyperhomocysteinemia: a complex interaction

**DOI:** 10.1186/s13098-017-0218-0

**Published:** 2017-03-21

**Authors:** Daniel E. Platt, Essa Hariri, Pascale Salameh, Mahmoud Merhi, Nada Sabbah, Mariana Helou, Francis Mouzaya, Rita Nemer, Yasser Al-Sarraj, Hatem El-Shanti, Antoine B. Abchee, Pierre A. Zalloua

**Affiliations:** 1 Bioinformatics and Pattern Discovery, IBM T. J. Watson Research Centre, Yorktown Hgts, NY 10598 USA; 2 School of Medicine, Lebanese American University, Chouran, Beirut, 1102 2801 Lebanon; 3 School of Pharmacy, Lebanese American University, Byblos, Lebanon; 4 Qatar Biomedical Research Institute, Doha, Qatar; 5 University of Iowa Carver College of Medicine, Iowa City, USA; 6 Division of Cardiology, Department of Internal Medicine, School of Medicine, American University of Beirut, P.O. Box: 11-0236, Riad-El-Solh, Beirut, 1107 2020 Lebanon; 7 Harvard School of Public Health, Boston, MA 02215 USA

**Keywords:** Homocysteine, MTHFR C667T, Diabetes mellitus

## Abstract

**Background:**

Elevated homocysteine (Hc) levels have a well-established and clear causal relationship to epithelial damage leading to coronary artery disease. Furthermore, it is strongly associated with other metabolic syndrome variables, such as hypertension, which is correlated with type II diabetes mellitus (T2DM). Studies on T2DM in relation to Hc levels have shown both positive and negative associations. The aim of the present study is to examine the relationship between Hc levels and risk of T2DM in the Lebanese population.

**Methods:**

We sought to identify whether Hc associates positively or negatively with diabetes in a case–control study, where 2755 subjects enrolled from patients who had been catheterized for coronary artery diagnosis and treatment. We further sought to identify whether the gene variant MTHFR 667C>T is associated with T2DM, and how Hc and MTHFR 667C>T also impact other correlates of T2DM, including the widely used diuretics in this study population.

**Results:**

We found that Hc levels were significantly reduced among subjects with diabetes compared to those without diabetes when adjusted for all potential confounders (OR 0.640; 95% CI [0.44–0.92]; *p* = 0.0200). The associations between Hc levels and other variates contradicted the result: hypertension associates positively with high Hc levels, and with T2DM. The MTHFR 667C>T only associated significantly with high Hc levels.

**Conclusion:**

These results suggest population-specific variations among a range of mechanisms that modulate the association of Hc and T2DM, providing a probe for future studies.

## Background

The prevalence of type II diabetes mellitus (T2DM) continues to increase worldwide [1, 2]. A recent study estimated the prevalence of diabetes in the Middle East to be 9.3% [3], already more elevated than worldwide estimates of 8.3% in 2014 [4], with a rapid emergence due to recent dietary changes [5]. Given the significant healthcare-related expenditures, identifying modifiable factors is essential for the prevention of diabetes. T2DM is known to be a complex and heterogeneous disease resulting from a set of interacting factors that can be genetic or environmental, each with a variable contribution to disease causation. Many cultural, dietary, behavioral, contextual and lifestyle factors are also primary determinants of risk for T2DM [6].

Recent studies have increasingly been showing interactions between plasma Hc levels and T2DM and its vascular complications [7–9]. Correlation with T2DM remains unconvincing however, with studies reporting variability in plasma Hc levels between people with diabetes and people without diabetes [10–14]. Hc is a sulfur-containing, toxic non-proteinogenic amino-acid biosynthesized from methionine. It is located at a branch-point of multiple metabolic pathways and is produced from methionine as a product of a number of transmethylation reactions [15]. The most common inherited disorder leading to hyperhomocysteinemia is the 5-methylenetetrahydrofolate reductase (MTHFR) polymorphism [16, 17]. Individuals with deficiencies of folic acid (B9), pyridoxine (B6), or cobalamin (B12) can as well develop hyperhomocysteinemia [18, 19].

To date, the mechanisms behind the T2DM correlation with Hc levels have been difficult to identify. MTHFR converts 5,10-methylenetetrahydrofolate to 5-methyltetrahydrofolate. Mutations in MTHFR impair the function of the enzyme, and the 677C → T polymorphism (MTHFR, MIM # 607,093) is an enzyme reducing activity variant associated with elevated plasma Hc levels as well as insulin resistance. [20–22]. It is believed that the decreased insulin secretory responsiveness, caused by the destructive production of reactive oxygen species (ROS) as a result of elevated Hc levels, leads to insulin resistance [23, 24]. It has also been suggested that in patients with insulin resistance, there is hepatic acceleration of glucocorticoid secretion that also leads to enhanced Hc catabolism and decreased plasma Hc levels [14].

Genetic modifiers of significant biological effect could promote our understanding of the mechanisms of action by which Hc levels and MTHFR variants contribute to T2DM. These mechanisms of interactions have not yet been comprehensively elucidated, and explanations of these interactions have been contradictory. The aim of the present study is to examine the relationship between Hc levels and risk of T2DM in a phenotypically well-characterized group of patients and controls. We further investigate whether a dose–response relationship exists in our study population by assessing the interaction between the MTHFR 677C>T and Hc levels on T2DM. The Lebanese population may offer some unique features due to the rapid emergence of T2DM in Lebanon.

## Methods

Subjects were recruited from several hospitals in Lebanon between May 2007 and June 2013. We developed a questionnaire to measure the impact of T2DM risk factors and collected family history after obtaining an informed consent from participants, as approved by the Lebanese American University Institutional Review Board (IRB). Annotations were coded from medical charts for data such as laboratory tests, prescribed medications, and presence of other clinical conditions. Venous blood samples were drawn in ethylenediaminetetraacetic acid (EDTA) tubes from 7709 subjects. A diagnosis of T2DM was made when a patient had a hemoglobin A_1_C (HbA_1_C) value of 48 mmol/mol (6.5%) or higher and/or by an ascertained physician supported by documentation in the patient’s medical records. DNA was extracted using a standard phenol–chloroform extraction procedure. The single-nucleotide polymorphism (SNP) rs1801133 from MTHFR was determined for 1824 of the 2755 study samples, having been analyzed using the Illumina Human610 and 660 W Quad beadchip and the Illumina Human Omni EXP-12v1 multi-use.

Plasma homocysteine level was determined for the 2643 subjects by a microparticle enzyme immunoassay (MEIA) method (Abbott). The assay was carried out following the instructions of the manufacturer. Hyperhomocysteinemia was defined as a concentration of ≥15 μmol/l, and elevated Hc level as ≥10 μmol/l. 1007 of these samples were genotyped. Dyslipidemia was defined as low high-density lipoprotein (HDL) (≤40 for men, 50 for women), high triglycerides (≥200), and diagnosis of hyperlipidemia (DxHL). Since most subjects had controlled low-density lipoprotein (LDL) with a protective effect against LDL levels ≥120 given a diagnosis of hyperlipidemia (OR 0.730, 95% CI = 0.60–0.89, *p* = 0.0014), elevated LDL levels were not included for analysis. Diagnoses of T2DM (DxT2DM) and hypertension (DxHTN) were also included. BMI in excess of 30 (obesity) was also analyzed.

Besides summary statistics (Table 1), we sought to identify dominating covariates associated with hyperhomocysteinemia. Noting T2DM negatively associated with hyperhomocysteinemia, we considered whether the structure of enrollment induced Berkson’s bias, and tested for interaction with enrollment categories. Prediction of T2DM by hyperhomocysteinemia was performed given adjustment variables, first by including only the adjustment variables, then including hyperhomocysteinemia and finally including interactions between hyperhomocysteinemia with DxHTN. The adjustment variable analysis provides a baseline to identify shifts due to inclusion of hyperhomocysteinemia and its interaction with DxHTN. Following that, the same analysis of T2DM was repeated except that hyperhomocysteinemia was replaced by elevated Hc levels. Finally, rs1801133 in MTHFR, known to promote hyperhomocysteinemia, was tested for its effects on other metabolic syndrome variables and for elevated Hc and hyperhomocysteinemia.

**Table 1:** Summary statistics, including counts of total participants by sex, coffee consumption, and diabetes diagnoses, and average (standard error of mean) for age, total cholesterol, HDL, LDL, Tg, Hc, and BMI levels

	Total	Male	Female
Count	7709	5188	2517
Age	61.2 (11.4)	60.2 (11.6)	63.4 (10.8)
Total cholesterol	183.5 (46.8)	180.4 (46.2)	190.0 (47.7)
HDL	39.8 (12.1)	37.5 (10.6)	45.0 (13.4)
LDL	111.0 (41.9)	110.1 (41.9)	112.8 (41.9)
Tg	178.5 (113.0)	181.5 (116.1)	172.1 (105.9)
BMI	29.2 (5.3)	28.6 (4.8)	30.4 (6.0)
HTN	4722	2899	1821
Diabetes count	2404	1551	852
Homocysteine	14.9 (8.0)	15.6 (8.6)	13.4 (6.4)

## Results

**Fig. 1:**
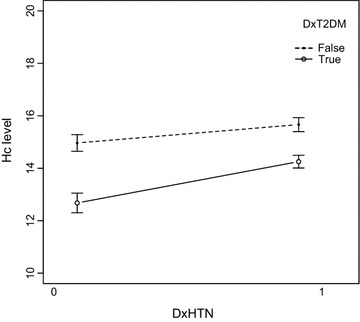
Homocysteine (Hc) levels showing interaction between hypertensive (DxHTN 1) and non-hypertensive (DxHTN 0) diabetic (DxT2DM True) and non-diabetic (DxT2DM False) subjects

Table 1 shows summary statistics for metabolic syndrome, and Hc for the study population. It is notable that males make up most of the population and the average Hc levels were either borderline or fully hyperhomocysteimic. Figure 1 shows the interaction between hypertension diagnosis and T2DM diagnosis on Hc levels in our study population. Hypertension diagnosis has a stronger impact on Hc levels among diabetics than among non-diabetics. The difference in Hc levels between diabetics and non-diabetics is clearly greater than the difference in Hc levels between hypertensive and non-hypertensive subjects.

We tested for the presence of Berkson’s bias by examining whether the association between Hc levels and T2DM interacted with subjects’ enrollment. There was no significant association between enrollment and elevated Hc levels (OR 0.870, 95% CI 0.72–1.05, *p* = 0.153), or hyperhomocysteinemia (OR 0.869, 95% CI 0.74–1.02, *p* = 0.0947). Table 2 shows very significant associations of hyperhomocysteinemia and elevated Hc with age and sex. Associations of hyperhomocysteinemia and elevated Hc with other variants were analyzed with and without adjustment with sex and age. T2DM is suppressed among subjects with elevated Hc with and without adjustment, but not among subjects with hyperhomocysteinemia. Hypertension is significantly promoted both by hyperhomocysteinemia and elevated Hc.

**Table 2:** Predicting clinical metabolic syndrome variates from homocysteine, both unadjusted, and adjusted with sex and age

	Homocysteine ≥ 10	Homocysteine ≥ 10 (adjusted)	Homocysteine ≥ 15	Homocysteine ≥ 15 (adjusted)
Age ≥ 60	1.405 (1.17–1.69) 0.000294		1.504 (1.28–1.77) 8.81 × 10^−7^	
Sex = male	2.074 (1.71–2.50) 2.98 × 10^−14^		1.586 (1.33–1.90) 3.23 × 10^−7^	–
DxHL	0.844 (0.70–1.01) 0.0706	0.873 (0.72–1.05) 0.154	0.816 (0.69–0.96) 0.0138	0.833 (0.71–0.98) 0.0286
HDL ≤ 40 (men), 50 (women)	0.952 (0.78–1.16) 0.628	1.014 (0.70–1.24) 0.890	1.085 (0.91–1.29) 0.356	1.151 (0.97–1.37) 0.115
Tg ≥ 200	0.977 (0.80–1.20) 0.826	0.968 (0.79–1.20) 0.764	0.923 (0.77–1.11) 0.390	0.937 (0.78–1.13) 0.491
DxT2DM	0.710 (0.59–0.86) 0.000521	0.698 (0.57–0.85) 0.000368	0.879 (0.74–1.05) 0.151	0.862 (0.72–1.03) 0.103
DxHTN	1.244 (1.03–1.50) 0.0212	1.301 (1.07–1.58) 0.00795	1.468 (1.24–1.74) 6.72 × 10^−6^	1.479 (1.24–1.76) 9.91 × 10^−6^
BMI ≥ 30	0.906 (0.75–1.10) 0.310	0.995 (0.82–1.21) 0.957	1.027 (0.87–1.21) 0.755	1.081 (0.91–1.28) 0.373

Each block reports OR (95% CI), and *p* value

Table 3A shows the impact of hyperhomocysteinemia on T2DM considering adjustments from the other covariates. Inclusion of the strongest covariates (age, sex included as adjustment variables but not reported), DxHL, obesity, and DxHTN were all highly significant. Inclusion of hyperhomocysteinemia tended to shift the other adjustment variable odds ratios marginally, with BMI losing high significance, yet remaining significant; hyperhomocysteinemia was significant. Inclusion of the interaction with hypertension was not significant, but hyperhomocysteinemia remained significant. Table 3B shows patterns similar to that of 3A except that elevated Hc levels had more pronounced OR values and were more highly significant.

**Table 3:** Predicting T2DM, adjusted by sex = male and age ≥ 60

A				
Homocysteine ≥ 15	DxHTN	DxHTN × homocysteine ≥ 15	DxHL	BMI ≥ 30
	2.058 (1.84–2.31) <2 × 10^−16^		1.573 (1.42–1.74) <2 × 10^−16^	1.335 (1.20–1.48) 5.37 × 10^−8^
0.810 (0.67–0.98) 0.0280	2.362 (1.94–2.88) <2×10^−16^		1.621 (1.36–1.93) 7.65 × 10^−8^	1.225 (1.02–1.46) 0.0258
0.640 (0.44–0.92) 0.0200	2.160 (1.72–2.72) 4.85 × 10^−11^	1.374 (0.89–2.13) 0.150	1.622 (1.36–1.93) 7.53 × 10^−8^	1.225 (1.02–1.46) 0.0259

B				
Homocysteine ≥ 10	DxHTN	DxHTN × homocysteine ≥ 10	DxHL	BMI ≥ 30
	2.058 (1.84–2.31) <2×10^−16^		1.573 (1.42–1.74) <2 × 10^−16^	1.335 (1.20–1.48) 5.37 × 10^−8^
0.664 (0.54–0.82) 0.00011	2.374 (1.95–2.90) <2 × 10^−16^		1.625 (1.36–1.94) 6.96 × 10^−8^	1.219 (1.02–1.46) 0.0301
0.628 (0.44–0.90) 0.00937	2.235 (1.55–3.25) 2.09 × 10^−5^	1.087 (0.70–1.67) 0.705	1.624 (1.36–1.94) 7.07 × 10^−8^	1.220 (1.02–1.46) 0.0295

Each block reports OR, (95%CI), and *p*-value

Table 4 summarizes the allele frequencies of rs1801133 in the study population, with Hardy–Weinberg disequilibrium almost significant. Table 5 repeats Table 2, except that Hc levels are replaced by rs1801133 in an additive logistic regression. All associations were not statistically significant except for those involving Hc. Hyperhomocysteinemia was highly significant (OR 1.483, 95% CI 1.20–1.83, *p* = 0.000237), with elevated Hc being only significant (OR 1.287, 95% CI 1.05–1.59, *p* *=* 0.0173).

**Table 4:** MTHFR667 (rs1801133) genotype and allele frequency counts, and Hardy–Weinberg Chi square test results

rs1801133	Frequency	Relative frequency
TT	233	0.1277
CT	784	0.4298
CC	807	0.4424
T	625	0.3427
C	1199	0.6573

Hardy–Weinberg $$\chi_{1}^{2} = 3.8358$$ *, p* = 0.0502

**Table 5:** Predicting clinical metabolic syndrome variates from rs1801133, both unadjusted, and adjusted with sex and age

	rs1801133 (allele T)	rs1801133 (adjusted)
Age ≥ 60	1.026 (0.90–1.18) 0.708	–
Sex = male	0.963 (0.83–1.12) 0.618	–
DxHL	0.996 (0.87–1.14) 0.948	0.995 (0.87–1.14) 0.942
HDL ≤ 40 (men), 50 (women)	1.028 (0.89–1.18) 0.706	1.028 (0.89–1.19) 0.699
Tg ≥ 200	0.946 (0.82–1.09) 0.447	0.951 (0.82–1.10) 0.499
DxT2DM	1.025 (0.89–1.19) 0.737	1.022 (0.88–1.18) 0.767
DxHTN	1.028 (0.90–1.18) 0.685	1.021 (0.89–1.17) 0.766
BMI ≥ 30	1.031 (0.90–1.18) 0.670	1.028 (0.89–1.18) 0.695
Homocysteine ≥ 15	1.481 (1.20–1.83) 0.000234	1.483 (1.20–1.83) 0.000237
Homocysteine ≥ 10	1.277 (1.04–1.57) 0.0188	1.287 (1.05–1.59) 0.0173

Each block reports OR (95% CI), and *p*-value

## Discussion

To our knowledge, this is the first study done on the Lebanese population to examine the correlation of homocysteine levels and T2DM. The mean Hc plasma levels in patients without diabetes in our study population (15.3 ± 8.7) was elevated and found to be higher than what has been reported in previous studies (range = 12–14 mmol/l) [25]. We observed a significantly negative correlation between hyperhomocysteinemia and T2DM. This negative correlation remained significant whether Hc plasma levels where ≥10 or ≥15, indicating a consistent threshold association. Given the correlation of T2DM and coronary artery disease (CAD), a previous study conducted on our study subjects showed an association of hyperhomocysteinemia with the degree of coronary artery stenosis in CAD patients [26].

Our results are not aligned with the overwhelming majority of previous studies that have reported a positive correlation between elevated Hc and T2DM and its complications [27–29]. A recent meta-analysis study conducted on more than 8000 subjects provided strong support for a causal association of elevated Hc levels and T2DM [30]. This meta-analysis study, however, used published data from various studies that may have had different patient recruitment criteria and clinical definitions with many including small number of subjects. Measurements of Hc levels were not homogenous across all populations with each having a distinct genetic background hence leading to significant heterogeneity. This data variability may explain some of the difference in their findings compared to ours. One study in particular, conducted on 105 subjects with diabetes and 120 controls matched for sex showed that the mean fasting Hc was significantly lower in patients with diabetes than control subjects [13]. A study conducted on Mediterranean patients with T2DM did not show a difference in Hc levels between diabetic and non-diabetic patients [31], while a study done by Russo et al. [32] did not show a difference between total Hc among diabetic and non-diabetic women, suggesting a possible gender effect on this association. Moreover, despite the strong evidence showing the causal association of Hcy level with the development of T2DM [30], the Prospective Investigation of the Vasculature study in Uppsala Seniors (PIVUS) cohort (n  =  1016) showed no evidence of a causal relationship of levels of plasma homocysteine with fasting glucose, fasting insulin, or T2DM [33].

The reduction of Hc levels among subjects with diabetes could possibly be attributed to two factors. First, Hc is located at a branch-point of multiple metabolic pathways and is produced from methionine as a product of a large number of transmethylation reactions [15]. Homocysteine methyltransferase (MTHFR) and cystathionine beta synthase (CBS) carry out a chemical reaction that converts Hc to methionine when Hc is methylated by N-5-methyltetrahydrofolate. This remethylation reaction is the main regulator of plasma Hc levels. Second, it was consistently shown that in rats with diabetes, the expression of CBS is significantly increased. Hc levels were significantly increased when these rats with diabetes received insulin. These results strongly suggest a regulatory role of insulin in the hepatic trans-sulfuration pathway that metabolizes Hc. In vitro studies conducted on cultured hepatocytes also demonstrated an increased activity of CBS [34].

It has been postulated that as modulators of Hc, MTHFR variants that inactivate MTHFR or lower its activity may be directly associated with increased risk of T2DM. Although previous studies have shown an association between MTHFR 677C>T and complications of diabetes like retinopathy [35, 36] and nephropathy [37], none could recognize this activity lowering variant as a risk factor for T2DM, and therefore the negative association that we observe remains questionable. Moreover, mild hyperhomocysteinemia and the MTHFR TT genotype were not shown to be significant risk factors for the development of microangiopathy in patients with T2DM in one prospective cohort study by Russo et al. [38]. This suggests that there are other confounding variables not yet identified that may be impacting Hc levels and T2DM, which are not related to MTHFR mutations. In a recent meta-analysis study, a link between MTHFR 677C>T and T2DM was established, showing that subjects with the T allele of the MTHFR 677C>T variant have significantly higher risk of having diabetes (OR 1.31, *p* = 0.032) than carriers of the C allele [30].

In our study we failed to show a direct association between the MTHFR 677C>T variant and T2DM. This suggests that there are other causes to hyperhomocysteinemia acting in our population that impact its association either directly or through high Hc levels with hypertension not accounted for by MTHFR 677C>T. We did not resolve an interaction between diuretics and MTHFR 677C>T in predicting hyperhomocysteinemia, but additive regression showed both contributed highly significant OR’s. Yet, MTHFR 677C>T shows no significant association with T2DM. One possible reason behind the difference observed from the various studies associating MTHFR with T2DM is the genetic heterogeneity of the various populations studied. The frequencies of the MTHFR 677 alleles vary significantly from one population to another. The T allele ranges from 8.8% in Moroccans [39] to 19.1% in Chinese [40] population while it was 34.3% in our study, which although yields increased power, but limitations in the number genotyped restrict power. A systematic review by Zhong et al. [41] showed that rs1801133 polymorphism of the MTHFR gene was not consistently associated with either increased or reduced risk of T2DM.

## Limitations

All enrolled subjects were catheterized. Catheterization was performed for myocardial infarction, unstable angina, and subjects warranting workup due to high CAD risk factors. We therefore tested whether the negative association between hyperhomocysteinemia and T2DM might be due to Berkson’s bias, and found that there was no association between reason for catheterization and the relationship between homocysteine levels with T2DM. Another limitation of the study is the absence of data related to levels of vitamins B9 (folate) and B12 or creatinine levels reflecting kidney functions; thus these parameters were not included in the final multivariate analysis despite being associated with homocysteinemia.

## Conclusion

We speculate that the role of MTHFR and their interaction with insulin and glucose levels in the blood shows mixed pressures on both positive and negative associations between Hc levels and T2DM, and the conditions reflected in populations producing these disparate results need to be further elucidated. The expression regulation of these enzymes is a complex process that may involve the activity of other enzymes as well as the cellular environment that is affected by diet, cellular stress and genomic background and the epigenetic milieu. The interaction between glucose metabolism, insulin resistance and the pathogenicity of hyperhomocysteinemia remains to be unraveled.

## Abbreviations

BMI: body-mass index
CAD: coronary artery disease
CBS: cystathionine beta synthase
DxHL: diagnosis of hyperlipidemia
DxHTN: diagnosis of hypertension
DxT2DM: diagnosis of type 2 diabetes mellitus
EDTA: ethylenediaminetetraacetic
HbA_1_C: hemoglobin A_1_C
Hc: homocysteine
HD: hemodialysis
HDL: high-density lipoprotein
LDL: low-density lipoprotein
MEIA: microparticle enzyme immunoassay
MIM: mendelian inheritance in man
MTHFR: 5-methylenetetrahydrofolate reductase
ROS: reactive oxygen species
SNP: single-nucleotide polymorphism
Tg: triglyceride
T2DM: type 2 diabetes mellitus
